# Genetic variations and gene expression profiles of Rice Black-streaked dwarf virus (RBSDV) in different host plants and insect vectors: insights from RNA-Seq analysis

**DOI:** 10.1186/s12864-024-10649-9

**Published:** 2024-07-30

**Authors:** Arezoo Lagzian, Abozar Ghorbani, Saeid Tabein, Roohallah Saberi Riseh

**Affiliations:** 1 Department of Plant Protection, Faculty of Agriculture, Vali-e-Asr University of Rafsanjan, Rafsanjan, Iran; 2 Nuclear Agriculture Research School, Nuclear Science and Technology Research Institute, Karaj, Iran; 3 Department of Plant Protection, Faculty of Agriculture, Shahid Chamran University of Ahvaz, Ahvaz, Iran

**Keywords:** RBSDV, RNA-Seq datasets, Genetic diversity, Host specificity

## Abstract

*Rice black-streaked dwarf virus* (RBSDV) is an etiological agent of a destructive disease infecting some economically important crops from the *Gramineae* family in Asia. While RBSDV causes high yield losses, genetic characteristics of replicative viral populations have not been investigated within different host plants and insect vectors. Herein, eleven publicly available RNA-Seq datasets from Chinese RBSDV-infected rice, maize, and viruliferous planthopper (*Laodelphax striatellus*) were obtained from the NCBI database. The patterns of SNP and RNA expression profiles of expected RBSDV populations were analyzed by CLC Workbench 20 and Geneious Prime software. These analyses discovered 2,646 mutations with codon changes in RBSDV whole transcriptome and forty-seven co-mutated hotspots with high variant frequency within the crucial regions of S5-1, S5-2, S6, S7-1, S7-2, S9, and S10 open reading frames (ORFs) which are responsible for some virulence and host range functions. Moreover, three joint mutations are located on the three-dimensional protein of P9-1. The infected RBSDV-susceptible rice cultivar KTWYJ3 and indigenous planthopper datasets showed more co-mutated hotspot numbers than others. Our analyses showed the expression patterns of viral genomic fragments varied depending on the host type. Unlike planthopper, S5-1, S2, S6, and S9-1 ORFs, respectively had the greatest read numbers in host plants; and S5-2, S9-2, and S7-2 were expressed in the lowest level. These findings underscore virus/host complexes are effective in the genetic variations and gene expression profiles of plant viruses. Our analysis revealed no evidence of recombination events. Interestingly, the negative selection was observed at 12 RBSDV ORFs, except for position 1015 in the P1 protein, where a positive selection was detected. The research highlights the potential of SRA datasets for analysis of the virus cycle and enhances our understanding of RBSDV’s genetic diversity and host specificity.

**Supplementary Information:**

The online version contains supplementary material available at 10.1186/s12864-024-10649-9.

## Introduction

*Rice black-streaked dwarf virus* (RBSDV), a member of the *Fijivirus* genus in the *Reoviridae* family, is a significant pathogen affecting rice and maize crops, causing black-streaked and rough dwarf symptoms, respectively [1–4]. The virus is transmitted by the small brown planthopper, *Laodelphax striatellus*, in a persistent propagative manner [5–10]. RBSDV consists of icosahedral, two-layered particles approximately 75–80 nm in diameter, with a genome consisting of 10 segments (S) of double-stranded genomic RNAs (dsRNAs) [11]. Each segment encodes specific proteins involved in various functions related to viral replication, structure, and pathogenicity [12–16]. The segments S1, S2, S3, S4, S6, S8, and S10 are harboring only an ORF which encodes a single protein (P) including RNA-dependent RNA polymerase (RdRp) [12], the major core structural protein [13], a protein with guanylyl transferase activity [14], the outer-shell B-spike protein, the viral RNA-silencing suppressor, the major capsid protein, and the outer capsid protein, respectively. Each of segments S5, S7, and S9 are encoded for two proteins [15, 17, 18] with different functions in the formation of viroplasm, tubular structures and viral genome replication [16].

Genetic analysis of RBSDV has revealed variations in different genomic segments and selective pressures acting on them. Structural proteins such as P2 and P4 show higher conservation compared to non-structural protein P9. The S9 genomic segment exhibits the highest nucleotide diversity, while the S10 segment contains the highest number of conserved regions. Furthermore, the previous report has indicated that S5-2 and S2 were under the highest and lowest selective pressure, respectively [19]. Several RBSDV proteins, such as P6, P7-1, P7-2, P9-1, and P10, are involved in viral pathogenesis and interaction with host factors [11, 20–22].

A viral species consists of populations of different mutants which are called “*quasispecies*” [23]. RNA viruses, including RBSDV, exhibit high mutation rates, which contribute to genetic diversity and evolution. Recombination, selection pressure, and genetic drift play significant roles in shaping the quasispecies structure of viral populations [24–27]. Understanding the rate and nature of these changes is crucial for developing effective strategies to control viral diseases [28]. Pathogen-host interactions also influence genetic diversity within viral populations to adapt to different hosts and tissues [29, 30]. Molecular diagnostic methods, such as next-generation sequencing (NGS), including RNA sequencing (RNA-Seq) and small RNA sequencing (sRNA-Seq), have provided valuable tools for studying plant viromes [31, 32]. For decades, the identification and diagnosis of plant viruses was limited to proteins-based immunological tests such as the ELISA method or sequencing nucleotide fragments by polymerase chain reaction (PCR). Due to the lack of easy access to the entire transcriptome viral populations, the genetic diversity and evolution of plant viruses remained unknown. The recent most advanced tools based on NGS, whole RNA sequencing, and Metagenomics have greatly helped in investigating expression levels and the genetic diversity of plant viruses. Analyzing viral transcriptomes from entire populations can unveil a hidden reservoir of mutations within viral communities, representing the final genetic variations before protein expression [33–37]. To this end, for the first time we used the transcriptomic datasets of the RBSDV to reveal the mutations and genetic variation that occurred in these populations and to compare expression levels in the segments in the plant and insect hosts.

In this study, we aimed to analyze Sequence Read Archive (SRA) datasets from various plant and insect hosts to investigate mutations and genomic variations within RBSDV populations. We examined protein changes resulting from mutations and estimated the frequency of variant forms. Additionally, we explored the expression levels of each genomic fragment within the viral transcriptomes of different hosts.

## Materials and methods

### RNA-Seq datasets from infected hosts

RNA-Seq datasets from infected hosts were acquired for this study. We identified and excluded some of the data with low coverage during analysis. A total of eleven RNA-Seq datasets were obtained from the SRA-NCBI database with good quality and suitable coverage for the virus genome, and these datasets were originally derived from Chinese RBSDV-infected rice, maize, and the viruliferous planthopper, *L. striatellus*, generated from 2017 to 2020. The specific sequence datasets used in the current investigation are documented in Table 1. These datasets were generated using the advanced Illumina HiSeq 2000–4000 techniques, which have proven to be highly effective in analyzing RNA sequences.

**Table 1:** Properties of RNA-Seq datasets from RBSDV infected hosts which were analyzed in the present study

Dataset	Host	RNA-Seq Instrument	Layout	Spots (M)	Size (Gbp)	GC (%)	More Explanation	Country/ region	Year	Accession Number
D1	Rice^a^	Illumina HiSeq 2000	SINGLE^c^	15.9	2.2	47	RBSDV-susceptible rice cultivar^h^	China/ Jiangsu	2017	SRX2653517
D2-1	”	”	”	17	2.3	47.6	“	“	”	SRX2730361
D2-2	”	*“*	”	31	2.4	-	“	“	”	SRX2730362
RBSDV1	Rice^b^	Illumina HiSeq 2500	PAIRED^d^	38.8	5.2	44.7	Asian cultivated rice^i^	China/ Hangzhou	2020	SRX8967824
RBSDV2	”	”	”	34.5	4.6	45.4	“	“	”	SRX8967825
RBSDV3	”	”	”	37.8	5.0	44.6	“	“	”	SRX8967826
b73 t1	Maize ^b^	Illumina HiSeq 2000	SINGLE^f^	11.3	1.0	56.2	*Zea mays* B73^j^	China/ Jinan	2018	SRX3785264
b73 t2	”	”	”	11.3	1.0	56.1	“	“	”	SRX3785263
RB MG1	*L. striatellus* ^*b*^	Illumina HiSeq 4000*	PAIRED^g^	49.2	6.1	44.3	From indigenous *L. striatellus* midgut^k^ tissue	China/ Haian	2020	SRX8604131
RB MG2	”	”	”	49.8	6.0	46.7	“	“	”	SRX8604132
RB MG3	”	”	”	44.7	5.3	45.9	“	“	”	SRX8604133

^a^. Other Source, ^b^. Transcriptomic Source, ^c^. Unspecified, ^d^. cDNA, ^f^. RANDOM, ^g^. Size fractionation Selection. *. ncRNA-Seq ^h^ Rice plants (*Oryza sativa* L. ssp. Japonica), cultivar Kangtiaowuyujing 3 (KTWYJ3) were planted in the fields of Jiangsu province, China and infected by indigenous *L. striatellus* [38], ^i^ cultivar wuyujing 7, sampled from Hangzhou, China [39], ^j^ Planted in the fields where had frequently happened severe RBSDV infection in the maize, collected from Jinan province, China [40], and ^k^ Indigenous *L. striatellus* (Fallen) were collected from Haian, China and reared on RBSDV-infected rice [41]

### Preparing transcriptomic reads

Preparing transcriptomic reads involved several steps to ensure high-quality datasets. Initially, the quality control (QC) of the reads was carefully examined. To optimize the transcriptomic datasets, the reads were trimmed using CLC Genomics Workbench 20, software provided by QIAGEN. This trimming process involved removing adapters, ambiguous nucleotides, and low-quality regions from the fastq datasets. Default parameters were used in CLC Genomics Workbench, where bases below 15 nucleotides, a maximum of 2 ambiguous nucleotides, and a Qscore of < = 5 were considered for trimming. Subsequently, the trimmed reads were mapped to the reference genome of RBSDV (accession numbers NC_003728-NC_003737) that encompasses all ten double-stranded RNA (dsRNA) genomic fragments. This mapping procedure allowed for aligning the transcriptomic reads to the corresponding locations in the RBSDV reference genome, facilitating further analysis and interpretation.

### De novo genome assembly and virus sequence annotation

De novo genome assembly and virus sequence annotation were performed using mapped reads. The reads were collected and utilized for de novo assembly, employing default parameters such as a word size of 20 and a minimum contig length of 200 nt. Subsequently, the obtained contigs were subjected to annotation using an open reading frame (ORF) finding tool in Geneious Prime 2022, a software package developed in the Netherlands specifically for this purpose. Furthermore, a new reference genome was generated from our data, enabling the discovery of single nucleotide polymorphisms (SNPs) within intrapopulation.

### Assessment of genetic diversity

To investigate the genetic diversity, the RNA-Seq datasets were utilized in this study, and the virus sequences generated from these datasets were employed as the reference genome. The CLC Genomics Workbench was employed for the analysis. The following thresholds were set: a minimum coverage of 2, a minimum variant frequency of 0.01, a maximum variant p-value of 10^− 6^, and a minimum strand-bias p-value of 10^− 5^. By using the Geneious Prime software, the impact of genetic diversity on translational changes was examined. This analysis included an exploration of polymorphism types, protein effects, variant frequency, coding sequence (CDS) positions, amino acid changes, codon changes, and variant p-values. Furthermore, the assessment of SNPs within the ORFs of the virus was conducted. For the visualization of SNPs on the 3D protein structures, the Protein Data Bank (PDB) was downloaded from the RCSB PDB database (https://www.rcsb.org) and integrated with the CLC Genomics Workbench.

### Analysis of Virus Gene expression

In order to assess the gene expression profile for each ORF, the RNA-Seq dataset was aligned to the reference genome using the CLC Workbench software, utilizing default parameters as part of the RNA-Seq analysis option. These parameters included a length fraction of 80%, a fraction similarity of 80%, and costs of 2 for mismatches, 2 for deletions, and 3 for insertions. The virus reference genome was transformed into a genome track and gene tracks. The mapping results were then utilized to calculate the transcripts per million (TPM) for each ORF. The read counts were averaged across the replicates that were used. Because all samples are from different studies and may have some differences such as using poly A fraction or total RNA, we normalized all samples using “Normalize expression value” tools in CLC genomic workbench software with the normalize method (“by totals”). Finally, the gene expression profiles were compared not only among all genomic fragments but also between different hosts.

### Recombination analysis of virus fragments from various hosts

This study employed Geneious Prime software to analyze recombination events between RBSDV genetic fragments isolated from three different hosts: rice, maize, and an insect vector. The analysis focused on the coding sequences of the viral genetic fragments.

### Selective pressure on viral segments in different hosts

To investigate the evolutionary pressures acting on the RBSDV genetic fragments in each host, the ratio of non-synonymous (amino acid-changing) to synonymous (silent) substitutions (dN/dS) was calculated. A high dN/dS value indicates positive selection favoring changes in the protein sequence. Conversely, a low dN/dS value suggests purifying selection eliminating detrimental mutations. A value close to 1 suggests neutral evolution, where mutations are neither beneficial nor detrimental. The Datamonkey online tool (https://www.datamonkey.org/*)* was utilized to perform this analysis. This tool employs a method called MEME (Mixed Effects Model of Evolution) to estimate substitution rates at each site within the segments. MEME can distinguish between positive selection, purifying selection, and neutral evolution acting on specific sites within the genetic fragments.

### Codon usage bias analysis in different hosts

The study also analyzed the codon usage patterns of the viral CDS from each host. Codons are the three-letter sequences in RNA that specify amino acids during protein synthesis. Organisms can exhibit a preference for certain codons for specific amino acids, even though multiple codons can code for the same amino acid (synonymous codons). This preference is termed codon usage bias. The analysis involved isolating the coding sequences from all three hosts (rice, maize, and insect vector) and converting them into a FASTA format file. Subsequently, R software (version 9 of RStudio) with specific sequence analysis packages was employed. The coRdon package [42] was used for sequence management and manipulation. The sequences were imported into R using the readSet function from coRdon. To ensure that only coding regions were included, the check_cds function was employed. Subsequently, codon frequencies were calculated using the count_codons function, which determines the number of times each codon appears in the datasets. Several metrics were then computed to analyze codon usage bias including, (1) Relative Synonymous Codon Usage (RSCU): This metric indicates how often synonymous codons are used compared to their expected usage based on random chance. (2) Codon Adaptation Index (CAI): This metric reflects the bias towards codons frequently used in highly expressed genes in a specific organism. (3) Effective Number of Codons (ENC): This value indicates the overall level of codon usage bias within a gene. A lower ENC suggests a stronger bias towards a preferred set of codons. (4) GC content and GC3S: These metrics represent the proportion of guanine and cytosine nucleotides in the coding sequences, with GC3S focusing specifically on the third position of each codon.

The analysis calculated descriptive statistics (mean, median, standard deviation, and range) for all these metrics (CAI, ENC, GC content, and GC3S). Finally, histograms were generated to visualize the distribution of these values across the entire dataset.

## Results and discussion

### Viral genome Assembly

The QC scores of RNA-Seq datasets were checked to ensure that the transcriptomic data were suitable for further analysis. In different RNA-Seq datasets, the number of short reads ranged from 15 to 99 million (reading depth). The GC content was between 40% and 50%, and all reads in each dataset had the same length (length distribution) (Table 1). Two factors, enriched 5-mers and nucleotide contributions, appeared to be normal [43]. The per-sequence analysis indicated that most datasets had 0% of the ambiguous base. However, the content was less than 0.08% in the MG1 dataset from *L. striatellus* and it was only 7% in a few base positions of the reads, being 0% in RBSDV1, 2, and 3 datasets from rice. Furthermore, the QC pre-base analysis revealed that all datasets covered the complete length of reads, with a few exceptions in certain nucleotides for MG1, MG2, and MG3 datasets from *L. striatellus*, which had 95% coverage (Table 2). The clean reads were then mapped to the RBSDV reference genome. The highest percentage of mapped reads, 7.21%, was obtained from the RBSDV1, 2, and 3 datasets from rice. Approximately 0.01–0.02% of the total bases were mapped to the viral reference genome.

**Table 2:** Characteristics of mapped and non-mapped reads of infected-RBSDV datasets to RBSDV reference genome

Datasets	Mapped reads	Non-Mapped reads	Total reads	Percentage of Non-Mapped reads (%)	Hosts
D1	1,288	15,910,835	15,912,123	99.99	Rice
D2-1	1,635	16,991,742	16,993,377	99.99	
D2-2	103,317	61,903,405	62,006,722	99.83	
RBSDV1 2 3^∗^	15,944,685	206,081,484	222,026,169	92.82	
RBSDV1	3,170,927	74,377,792	77,548,719	95.91	
RBSDV2	3,998,481	64,900,125	68,898,606	94.20	
RBSDV3	8,775,277	66,803,567	75,578,844	88.39	
B73 t1	1,466	22,294,154	22,295,620	99.99	Maize
B73 t2	3,948	22,366,973	22,370,921	99.98	
BP MG1	20,696	98,423,154	98,443,850	99.98	Planthopper
BP MG2	65,301	99,630,142	99,695,443	99.93	
BP MG3	18,273	89,349,308	89,367,581	99.98	

∗ Three data that are duplicates were also mapped to each other

**Fig. 1:**
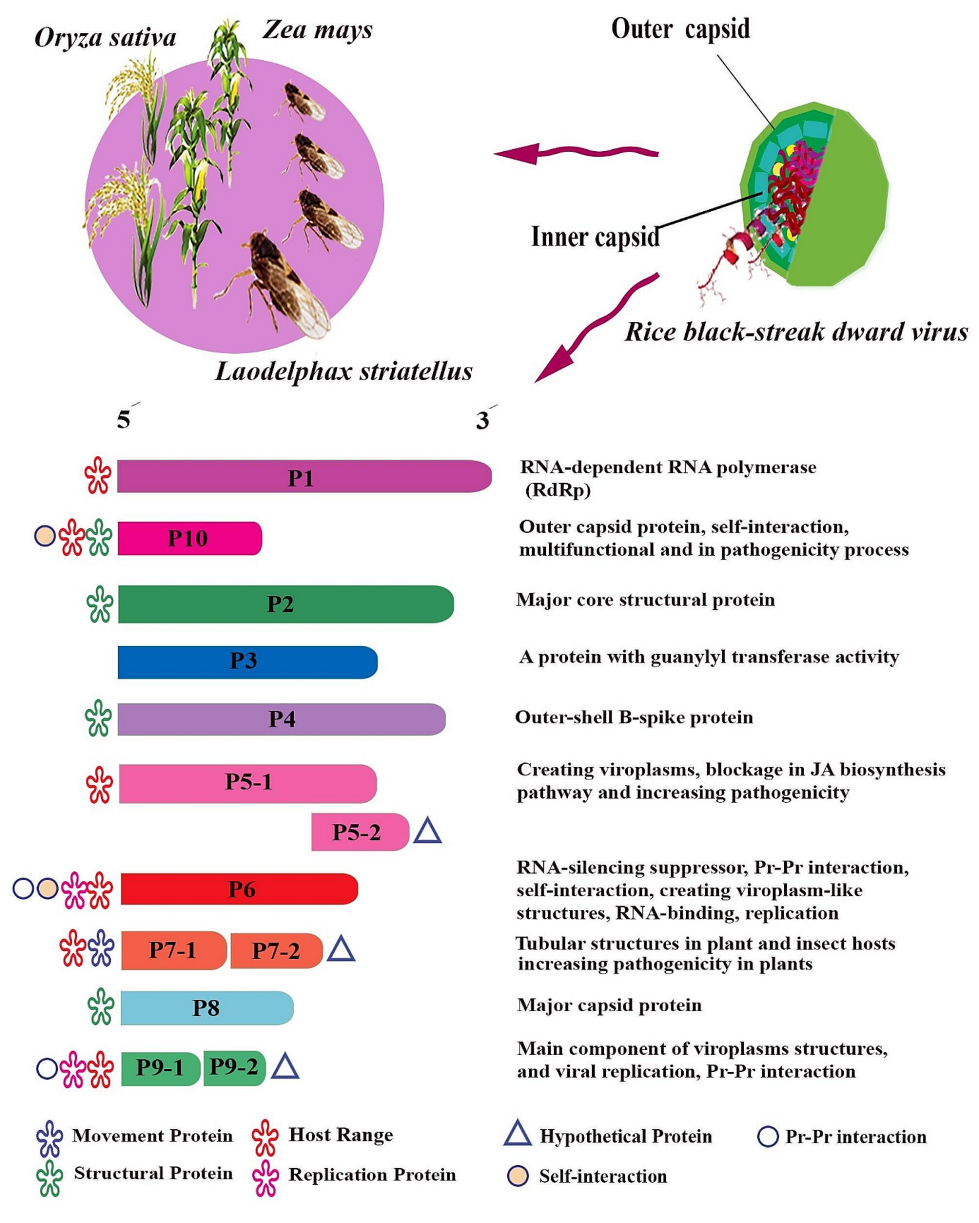
Schematic representation of Rice black-streaked dwarf virus genome. RBSDV genome consists of ten double-stranded fragments encoding 13 proteins with different functions. Pr: Protein. JA: Jasmonic acid

The result showed that the virus isolates in the present study had a genetic organization typical of the reference genome (Fig. 1). The genome of the Rice black-streaked dwarf virus consists of ten dsRNA molecules and each genomic fragment includes one or two segments [44, 45]. The virus ORFs encode 13 structural and non-structural proteins which play their role in the pathogenicity, virus replication, and the construction of viroplasms and tubular structures in the insect and plant host cells [16], (Fig. 1).

### Genetic Diversity of RBSDV in different hosts

To ensure more reliable results in investigating the polymorphism within replicative virus populations across different hosts, we combined multiple repeats of SRA datasets obtained from each host, including 6, 2, and 3 datasets obtained from RBSDV-infected rice and corn, and the viruliferous planthopper (Table 1), respectively. Subsequently, two crucial factors were evaluated: the frequency of variants and the conserved/influential regions during the virus cycle that affect the pathogenic cycle and host range in each ORF. We analyzed mutations that had codon-changing effects, such as substitutions, frameshifts, or deletions of the start codon. We specifically focused on mutants with a high frequency of variants and mutations occurring in genomic regions with critical functions in virus pathogenesis. We identified forty-seven mutated hotspots that co-occurred in numerous datasets with a high frequency of variants (HFV) (Table 3). Other mutants located in important regions did not necessarily co-occur in the datasets or exhibited a lower variant frequency (Supplementary 1). A total of 2646 mutations were associated with codon and protein shifts across the datasets (Supplementary 2).

All of the recognized SNPs with HVFs are localized in the important regions of S5-1, S5-2, S6, S7-1, S7-2, S9-1, and S10 ORFs [19, 20, 22, 46–49] (Table 3). The results showed that many single nucleotide polymorphisms with substitution protein effects (SPE) occurred in the replicative populations in different hosts, with a high frequency of variants. Many recognized SNPs were able to change codons and subsequently encode proteins with different variant frequencies. The RBSDV-susceptible rice cultivar KTWYJ3 datasets (D1, D2-1, and D2-2) contained the greatest number of hotspot mutants. In comparison, the datasets related to the RBSDV-infected rice cultivar Wuyujing 7 (RBSDV1, RBSDV2, and RBSDV3) had a lower frequency in the number of hotspot mutants. The mutated hotspots were abundantly found in the indigenous *L. striatellus* midgut of China (Haian) datasets (RB MG1, RB MG2, and RB MG3). The mutation in RBSDV-infected maize (*Zea mays* B73) datasets (b73 t1 and b73 t2) was more common in the hotspot of proteins P6, P7-2, P7-1, and P10, respectively. Furthermore, a total of thirty-two SNPs were detected in at least two different hosts, and five special SNPs were only recognized in *L. striatellus* (Table 3). Therefore, an accumulation of mutations in crucial regions of RBSDV was observed in the plant host cultivars and the native Chinese planthopper. Mutation, recombination, and reassortment are the primary forces driving genetic variation in viruses. RNA viruses and reverse transcribing (RT) viruses generally exhibit higher mutation rates (10^-6–10^-4 substitutions per nucleotide per cell infection) compared to double-stranded or single-stranded DNA viruses (10^-8–10^-6) [50–54]. This elevated mutation rate in RNA and RT viruses stems from the error-prone nature of their RNA-dependent RNA polymerase and RNA-dependent DNA polymerase (retrotranscriptases, RT), which lack proofreading and base excision repair mechanisms [55]. The transfer of newly introduced and indigenous viral species to native cultivars in a new area is one of the management challenges of viral diseases. Tomato yellow leaf curl disease (TYLCD), which is economically the most important viral pathogen in tomatoes, has been endemic in the Middle East. TYLCD gradually spread to Jordan and Iran through transmission from native infected hosts into new tomato cultivars [56]. Worst of all, TYLCD created severe pathogenicity in new variants due to the accumulation of mutations, recombination, and reassortment during the dissemination process [57]. Moreover, plant viruses can have genetic diversity in different cultivars of a plant. The transcriptomics analysis of indigenous and introduced potato cultivars revealed genetic diversity in the sequences of PVM, PVY, PVH and PVS viruses; and represented a heterogeneous distribution of the presence of pathogens in indigenous and introduced cultivars. More interestingly, a higher accumulation of single nucleotide polymorphisms was estimated in the underground tissues of the potatoes [30].

**Fig. 2:**
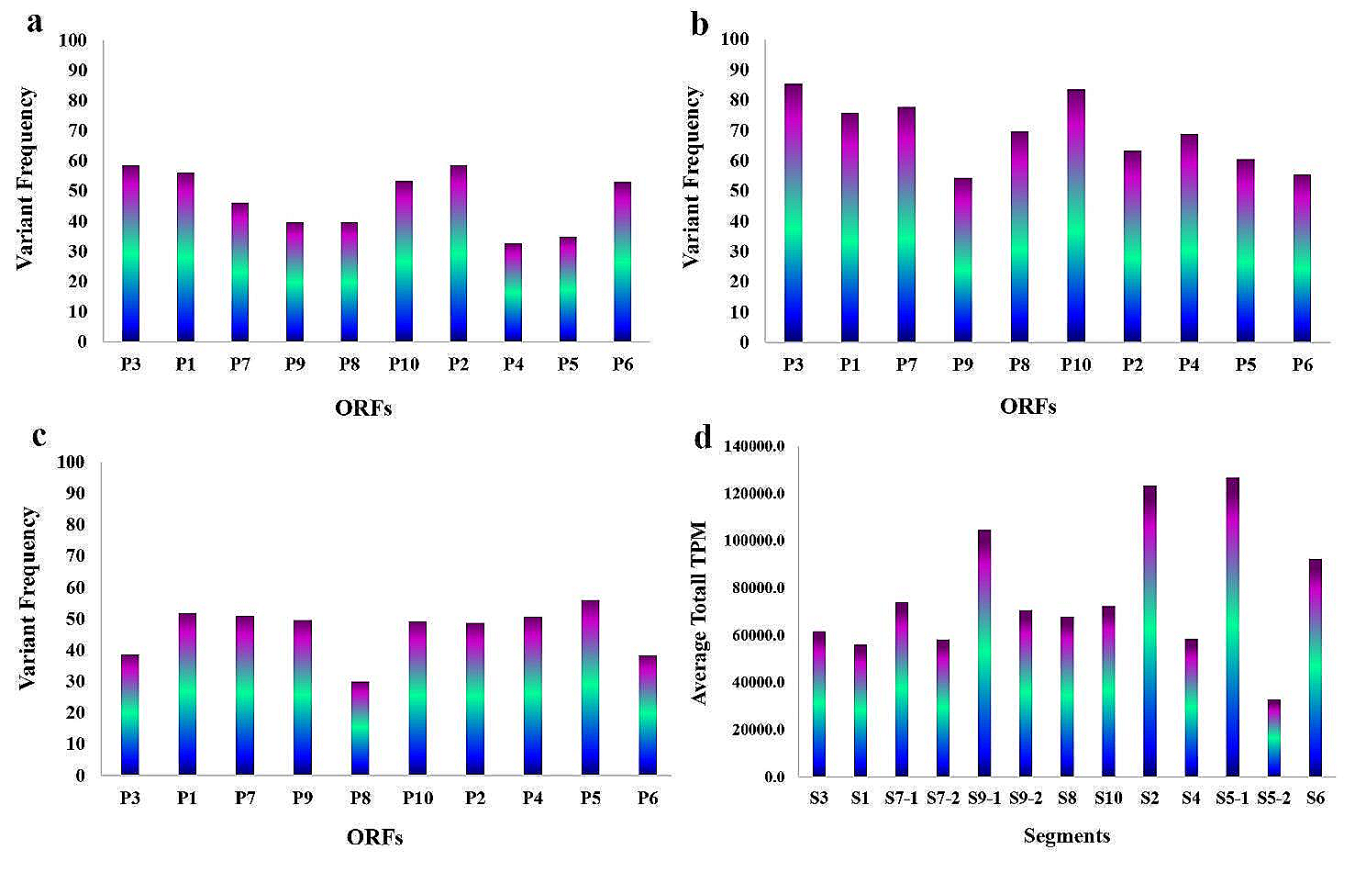
Average variant frequency within different ORFs in three hosts, rice (**a**), maize (**b**) and viruliferous planthopper (**c**). **d**. Average total Transcripts Per Million (TPM) in RBSDV genomic fragments using the CLC Workbench software

The average VF percentage was calculated for the SNPs that caused amino acid changes in genomic fragments. In rice, the highest VF was observed in the following genomic fragments: P2 (58.7%), P3 (58.6%), P1 (56.2%), and P10 (53.4%). In maize, the genomic fragments P3, P10, P7, and P1 showed the highest VF with average percentages of 85.4%, 83.6%, 77.8%, and 75.8%, respectively. In *L. striatellus*, the genomic fragments P5, P1, P7, and P4 had average percentages of 56%, 51.9%, 51%, and 50.6%, respectively, indicating the highest VF in these species (Fig. 2a, b, and c).

Therefore, the presence of all detected SNPs within the infecting virus populations of the planthopper showed more genetic diversity and, consequently, a greater possibility of an evolution for RBSDV in this host. Significant SNPs with HVF were detected in the S5 genomic fragment in different datasets from rice and *L. striatellus* hosts. The SNPs, including R146.7HP5 (in 2817 bp), M40.7TP5 (in 2499 bp), P9.7LP5 (in 2406 bp), S115.7NP5 (in 2724 bp), and T18.7IP5 (in 2433 bp), were recognized in the overlapping region of S5-1 and S5-2. Additionally, P228.7LP5 and A212.3TP5 appeared in a conserved region of S5-2 (Table 3). The S5 genomic fragment has the greatest number of RBSDV genomic conserved regions, including three highly conserved regions: 1-144 nt positions (5’ UTR), 2398–2832 nt position (overlapping region), and 3001–3164 nt position (3’ UTR). The nucleotide position between 2398 and 2832 nt is an overlapping region for S5-1 and S5-2, which showed lower genetic diversity compared to other regions of the S5 genomic fragment. This region plays an important role in the genetic diversity and evolution of the S5 genomic fragment [19, 47, 49, 58].

The SNPs, including I651.3VP6, Y581.7CP6, V572.3IP6, A545.3TP6, N492.7TP6, T583.3AP6, N516.3DP6, F518.3LP6, S509.3IP6, S473.3AP6, N510.3DP6, G587.7DP6, Q519.7LP6, S458.7NP6, N656.7TP6, and A655.7VP6, were detected at amino acid positions between 395 and 659. Two other SNPs, Y429.3PP6 and Y429.7SP6, were recognized at positions 404 to 439 of the S6 encoded protein. The P6 protein functions as an RNA silencing suppressor and is capable of forming viroplasm-like structures (VLSs) through self-interaction [20], and interaction with the P9-1 protein to create VLSs and possibly participate in viroplasm nucleation and virus morphogenesis processes [11]. The amino acid domain from 395 to 659 plays a crucial role in P6-P6 self-interaction and viroplasm formation in insect and plant hosts. Additionally, the domain from amino acids 404 to 439 plays a significant role in RNA-binding and viral replication [20]. The S7 segment consists of two ORFs, S7-1 and S7-2. The SNPs R294.3GP7, F108LP7, K302.7RP7, I301.7TP7, L300.3MP7, I298.7NP7, V297.3IP7, Q112.7LP7, N123.3DP7, and T294.3AP7, with variable VF ranging from approximately 6 to 74%, were identified in the SRA-data S7-1 of rice, maize, and planthopper (Table 3). The protein P7-1 induces the formation of tubules in the cells of planthoppers and plants, facilitating the spread of RBSDV within the organs. The tubular protein P7-1 is a critical factor responsible for the movement of RBSDV virions between cells [46, 47]. In insect cells, protein P7-1 facilitates the spread of the virus from midgut epithelium into visceral circular muscle through basal lamina which the dissemination has a mechanism similar to Southern rice black-streaked dwarf virus (SRBSDV), another member of the genus *Fijivirus* in the family Reoviridae. [18, 22]. A previous study demonstrated that the S7-1 protein encoded by SRBSDV contains two transmembrane domains located between amino acids 108 to 126 and 286 to 303 [59]. Despite the serological and pathogenicity similarities, the nucleotide similarity between RBSDV and SRBSDV is less than 78% [60]. Therefore, the hypothesis suggests the similarity of S7-1 domains between these two viruses. To test this hypothesis, the S7-1 amino acid sequences of both RBSDV (accession numbers NC_003730.1 and AJ297427) and SRBSDV (accession numbers NC_014710.1 and JQ692578) were aligned with each other.

Their amino acid identity with a query cover of 100% was 80.39%, and the expected domains at positions 108 to 126 and 286 to 303 amino acids showed 89% and 78% similarity, respectively. We identified a deletion of five amino acids (= 15 nucleotides) outside the two transmembrane domains in the S7-1 encoded protein (from amino acids 341 to 345) of SRBSDV. In contrast, Zhou et al. previously reported the removal of only 8 nucleotides from the S8 segment of SRBSDV compared to RBSDV [61]. Given the high identity between the two protein sequences and their similar function in host tubule formation, we considered the possibility of these two domains being important in RBSDV and checked for SNPs in these domains (Table 3). In S7-2, the SNPs E293.3KP7, I166.3LP7, R52.3CP7, G5.3SP7, Q288.7RP7, N149.7SP7, and S195.3RP7 occurred with high variable frequency (HVF) in all three hosts. The N-terminal region of ORF S7-2 is important which interacts with OSGJD2 and ZeaGID2 in plants [21]. The S10 viral genomic fragment in rice, maize, and the viruliferous planthopper exhibited high variant frequency SNPs. Specifically, C256.3SP10 in the conserved region of TM2, L124.3FP10 in the conserved region of TM1, and S68.7LP10, F108.0LP10, Y88.7CP10 in the N-terminal region. The RBSDV P10, a major external capsid protein with 558 amino acids and a molecular weight of 60 kDa, demonstrates multifunctionality in its interactions with both viral and host factors during viral infection. This protein is also known as integral membrane protein which causes stress in the endoplasmic reticular (ER) and consequently, the pronounced protein responses (for example, the activity of an inhibitor of actin polymerization) appear in plants [11, 22]. Previous studies have reported the presence of three conserved transmembrane domains: TM1 (119 to 137 aa), TM2 (250 to 270 aa), and TM3 (480 to 500 aa) in the S10 encoded protein [48]. Additionally, the N-terminal region of the S10 viral genome fragment, spanning amino acids 1 to 270, plays a crucial role in interacting with amino acids in LSRACK1 of the small brown planthopper, preventing RBSDV accumulation in cells [22]. Other studies showed that mutations in the Pepper mild mottle virus (PMMoV) coat protein can reportedly overcome *L*-gene resistance in pepper [62]. Moreover, for a virus to thrive through horizontal transmission by insect vectors, it needs a smooth two-step: efficient acquisition by the insect vectors and successful transmission to a new host. If a virus is acquired but struggles to be transmitted further, its spread within the plant population is severely limited [63]. Fascinatingly, viruses can manipulate their plant hosts in various ways. They might induce the production of specific morphological features or alter plant phenotypes, making them more attractive to insect vectors [64]. This, in turn, increases the chance of the virus reaching a new host. However, the exact mechanisms of how viruses manipulate insect vector selection and influence their dissemination success are complex [65, 66]. These manipulations have significant evolutionary implications [67]. The insect vector feeding preferences play a crucial role, as they determine the types of hosts the virus encounters. This shapes the virus’s evolutionary trajectory, pushing it towards becoming a specialist or a generalist pathogen [7].

**Fig. 3:**
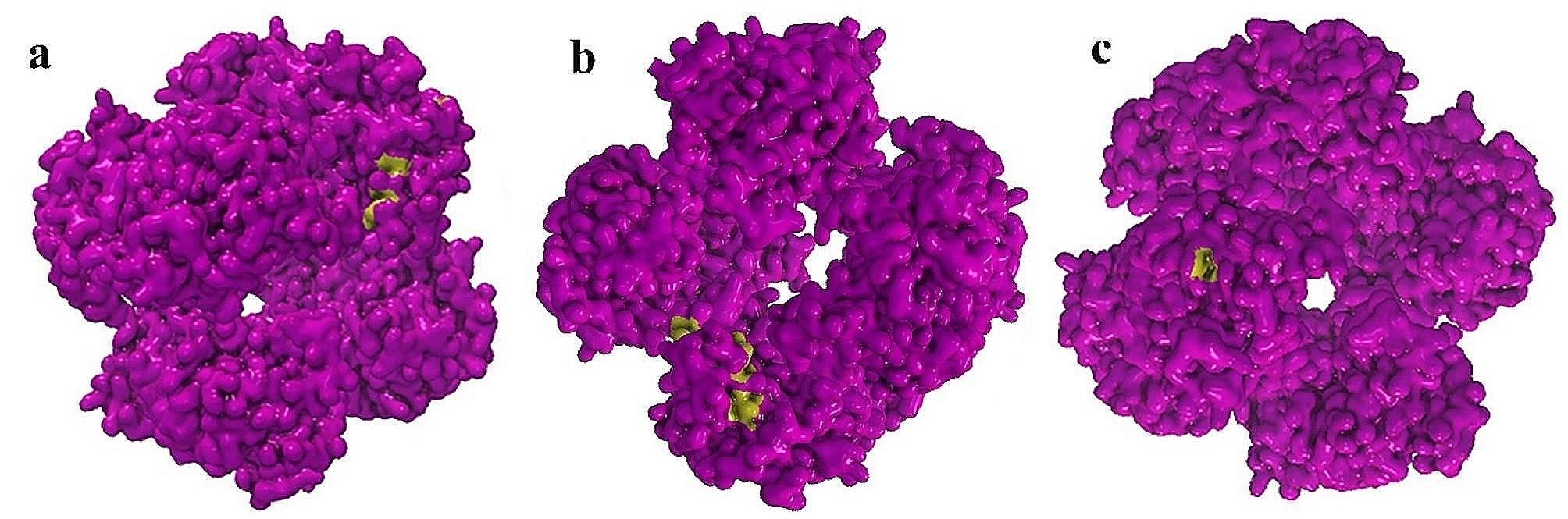
The mutations on the three-dimensional protein of P9-1 in amino acids. The reference amino acids (aa) have been shown with purple color and mutation positions have been shown in green color. **a**. mutation in aa position 168. **b**. mutation in aa position 20. **c**. mutation in aa position 84. Analysis was done by connecting to the PDB database

To gain a better understanding of the molecular implications of the identified genetic variations on the three-dimensional (3D) structure of proteins, we retrieved the PDB database and mapped the amino acid changes onto the RBSDV 3D proteins. Only mutations related to the P9 protein’s 3D structure could be linked to the PDB database. Numerous predicted protein changes were observed in various regions of the P9 protein, but the most significant alterations occurred at positions 168, 84, 20, 32, 151, 295, 104, 137, and 138 aa in the P9-1 regions (Fig. 3; Table 3; Supplementary 3). A previous study demonstrated that these amino acids are involved in the interaction between P9-1 and the viral P6 protein. Yeast two-hybrid assays revealed that even minor changes in the amino acids 1-347 of the P9-1 protein can disrupt the P9-1/P6 interaction and hinder replication processes [20].

Therefore, in total, the functions of pathogenicity and host range are carried out by the aforementioned encoded proteins P5, P6, P7, P9, and P10, each of which has conserved and effective regions for performing their functions [19, 20, 22, 46–49]. The RBSDV genome segments exhibited many mutations (2,646) with codon changes and different VFs. Approximately forty-seven co-mutated hotspots were identified in the important genetic regions of fragments P5-1, P5-2, P6, P7-1, P7-2, P9-1, and P10 through datasets, representing an extensive genetic resource for future changes of this virus in related functions. Most of the significant hotspots with high frequency were found in the populations of several hosts and datasets, indicating their rapid spread in RBSDV populations and serving as the likely reason for the formation of dominant populations. RBSDV is endemic to East Asian countries, such as China. The disease occurs in intermittent epidemic processes, making forecasting difficult [16]. Therefore, it is predicted that the mutated genetic resources will be frequently replicated in an epidemic with a massive reproduction rate.

The rice cultivar KTWYJ3 (RBSDV-susceptible) and indigenous *L. striatellus* datasets had the highest number of hotspots and the highest number of identical hotspots. Additionally, seven hotspots were observed in crucial regions of proteins exclusively in the *L. striatellus* datasets (including P5-2, overlapping P5-1 and P5-2, P6, P7-1, and P7-2). These findings suggest the potentially high sensitivity of the indigenous *L. striatellus* to RBSDV and highlight the high genetic diversity in these two types of RBSDV-infected populations: the rice cultivar KTWYJ3 and the Chinese indigenous *L. striatellus*. Moreover, some significant SNPs were identified on the 3D protein of P9-1 in RB MG1, RB MG2, RB MG3, D2-2, RBSDV1, RBSDV2, RBSDV3, and b73 t2 datasets.

Although the rate of genetic changes within the genome of RNA viruses is 104–107 times higher than that of their hosts, the virus’s ability to form pathogenicity and adapt to the host can be influenced by the surrounding environment [23, 68]. Mutations serve as valuable resources in populations, potentially facilitating virus transmission to new insect vectors or hosts [69, 70].

In comparison, we observed a smaller number of significant mutants in the rice cultivar Wuyujing 7 (RBSDV1, RBSDV2 and RBSDV3 datasets). However, these mutated hotspots also appeared in several other hosts. The presence of pro-viral host factors enables the virus to replicate and spread throughout the entire host [71, 72]. These pro-viral host factors can transform a tolerant host into a susceptible one. Host susceptibility depends on the balance between pro-viral host factors and suppressive responses. Any alteration in this balance leads to varying degrees of sensitivity to the virus [73–76]. Studies suggest that host factors are influenced by competition between host species and impact pathogenicity evolution [77]. For instance, the increase in pathogenicity of the TYLCD virus during its transfer to new tomato cultivars and its spread from the Middle East to the East was attributed to the accumulation of mutations, recombination, and reassortment [56, 57]. Mutations introduce genetic variation, which serves as the raw material for evolution and adaptation [78–81]. Laboratory studies reveal a fascinating interplay between plant RNA viruses and host immune mechanisms. Deficiencies in different host defense pathways significantly influence the rate of viral evolution, the types of genetic adaptations that emerge, and even the level of specialization the virus develops [82]. Adapting to specific host defenses is a complex challenge for viruses, as the host’s genetic makeup plays a crucial role in shaping the evolutionary arms race [83]. Furthermore, plant populations exhibit a remarkable heterogeneity in their defense responses, ranging from tolerance to susceptibility [84, 85]. This variation in host defenses plays a significant role in shaping the patterns of viral evolution, driving the emergence of various viral strains.

### Viral gene expression

**Fig. 4:**
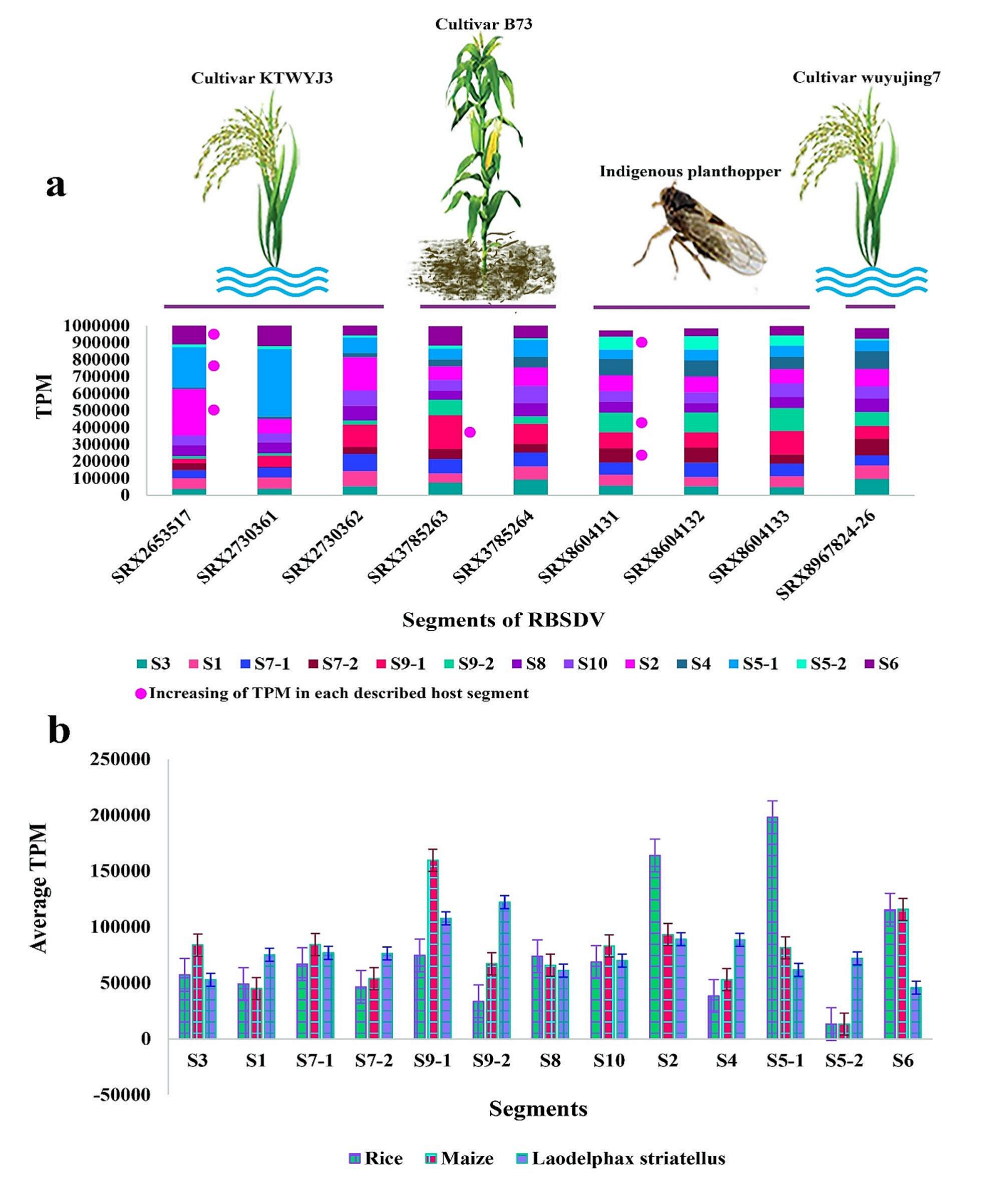
(**a**) Expression level of RBSDV genomic segments within SRA-sequences datasets (Table 1). (**b**) The expression level of RBSDV genomic segments in average data in each host. TPM: Transcript per million. The RNA-Seq dataset was aligned to the reference genome using the CLC Workbench software

The analysis of transcripts revealed distinct gene expression profiles for each dsRNA genomic fragment and ORF in various plant and insect hosts (Fig. 4). In infected rice (SRX8967824-26, SRX2653517, and SRX2730361-62 SRA datasets), the S5-1 ORFs had 198,042 reads, while S2 had 164,028 reads, and S6 had 115,470 reads, resulting in the highest TPM values. In maize (SRX3785263-64 SRA-sequences datasets), the ORFs with the highest TPM values were S9-1 (159,627 reads), S6 (115,844.47 reads), S2 (93,263 reads), and S7-1 (84,369 reads). The viruliferous planthopper dataset (SRX8604131-33 SRA dataset) showed the highest TPM values for the S9-2 (122,252 reads), S9-1 (107,783 reads), S2 (89,137 reads), and S4 (88,672 reads) ORFs (Fig. 4b). The number of reads in S1 49,125/ 45,074/ and 75,273 reads, and S3 57,270/ 83,979/ and 52,914 reads, and S8 73,914/ 65,844/ and 61,033 reads have been in rice/ maize/ and insect hosts, respectively.

The average TPM values were calculated to determine the transcription levels of genomic fragments in different hosts. The ORFs S5-1, S2, S9-1, and S6 exhibited the highest transcription levels with read numbers of 126,689.2, 123,338.8, 104,608.3, and 92,363.8, respectively (Fig. 2d). While the genomic fragments with the highest expression levels were similar in rice and maize, the expression levels of ORFs changed after entering the insect vector. The gene expression profile showed that S4, S5-2, and S9-2 ORF in both RBSDV-susceptible rice cultivar KTWYJ3 and maize datasets, as well as S5-2 ORF in rice cultivar wuyujing 7 datasets, exhibited low expression levels (or TPM). In contrast, the viruliferous planthopper datasets showed more expression uniformity, with increased expression levels observed for S5-2, S9-2, and S7-2 ORFs compared to other hosts (Fig. 4a). In both host plants, the highest expression levels were observed for S5-1, S2, S6, and S9-1 ORFs. In the insect vector, however, the highest expression levels were detected in S9-2, S9-1, S4, and S2 ORFs. The S2 ORF encodes the major core structural protein [4, 13]. The P5-1, P6, and P9-1 ORFs are responsible for producing viroplasm inclusions involved in RBSDV replication and assembly [86–88]. Although the functions and interactions of RBSDV-encoded proteins remain unclear, our findings suggest that S5-2, S7-2, and S9-2 may play an important role in virus/*L. striatellus* interactions.

### The recombination events and selection pressure

**Fig. 5:**
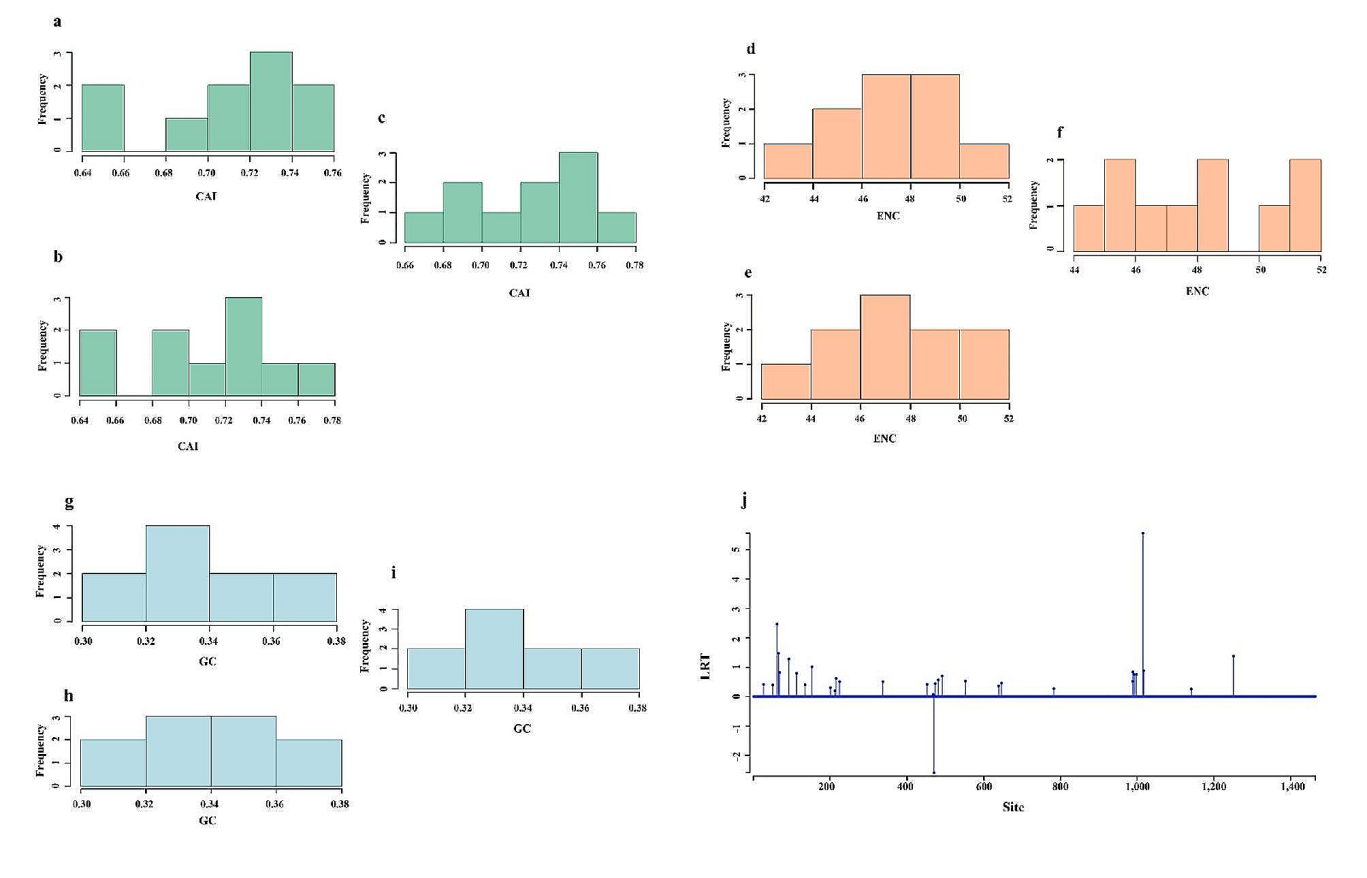
**a**-**c**. Histogram of Codon Adaptation Index (CAI) in *L. striatellus*, Rice and maize hosts, respectively. **d**-**f**. Histogram of Effective Number of Codons (ENC) in *L. striatellus*, Rice and maize hosts, respectively. **g**-**i**. Histogram of GC in *L. striatellus*, Rice and maize hosts, respectively. **j**. Likelihood Ratio Test (LRT) chart shows a positive selection of the P1 protein of RBSDV in CDS position site 1015

Our analysis showed no recombinant was found in all fragments of the virus genome. For the first time, it appears that protein P1 has a positive selection site (CDS position site:1015) with a p-value of 0.03 and a likelihood ratio test (LRT) value of 5.56. This suggests that there is a site in protein P1 that is under positive selection pressure. Positive selection pressure occurs when mutations are beneficial to the organism and are therefore favored by natural selection. This can lead to the rapid evolution of the gene [89]. The LRT chart shows the LRT values for each site across the genes. A higher LRT value indicates a stronger signal for positive selection. Overall, the analysis suggests that most of the RBSDV genes are under purifying selection pressure in different hosts, which means that mutations are being selected against. However, there is evidence of positive selection pressure at one site in protein P1. More analysis would be needed to determine the specific function of this gene and the role of the positively selected site (Fig. 5j). Previous studies showed that RBSDV displays a lower frequency of recombination events compared to some other viruses [90]. Moreover, the 13 RBSDV ORFs had already been showing that were under a negative selection (Ka/Ks < 1) [19].

### Codon usage bias

Based on the analysis, the effective number of codons (ENC) values are similar across the three hosts (*L. striatellus*, maize, and rice). The ENC values range from 0.20 to 0.40 for all three hosts. This suggests that there is a similar level of codon usage bias within genes across all three hosts (Fig. 5d-f). The codon adaptation index (CAI) values appear to be higher in rice compared to *L. striatellus* and maize. The CAI values for rice range from 0.60 to 0.80, while the CAI values for insect vector and maize range from 0.40 to 0.60. A higher CAI value indicates a stronger bias towards codons that are frequently used in highly expressed genes. This suggests that genes in rice may be more codon-optimized for translation than genes in *L. striatellus* and maize (Fig. 5a-c).

The GC (GC content) and GC3S (GC content at synonymous third positions) values also appear to be higher in rice compared to *L. striatellus* and maize. The GC and GC3S values for rice range from 0.50 to 0.70, while the GC and GC3S values for insect vector and maize range from 0.30 to 0.50. This suggests that rice may have a higher overall GC content and a higher proportion of G and C nucleotides at synonymous third codon positions compared to insect vectors and maize (Fig. 5g-i). Overall, the results suggest that there may be some differences in codon usage bias among the three hosts. Rice appears to have a higher CAI and GC content compared to *L. striatellus* and maize, suggesting that genes in rice may be more codon-optimized for translation. However, the ENC values are similar across all three hosts, suggesting that there is a similar level of codon usage bias within genes.

### Comparison of RSCU in Rice, Maize and *Laodelphax striatellus*

Supplementary 4, shows the relative synonymous codon usage (RSCU) values for all genes of virus isolated from three hosts: rice, maize, and *L. striatellus*. RSCU is a measure of codon bias in a gene, indicating how frequently synonymous codons are used compared to the expected usage if all codons were used equally. A value of 1 in the RSCU (Supplementary 4) indicates no bias, values greater than 1 indicate a positive bias (codon preferred), and values less than 1 indicate a negative bias (codon disfavored).

### The comparison for some amino acids

Phenylalanine (Phe): All three hosts show a preference for TTT codon over TTC. Rice and *L. striatellus* have a stronger bias towards TTT compared to Maize. Leucine (Leu): All three hosts show a preference for CT codon families (CTA, CTG, CTT) over TT codon families (TTA, TTG). Rice has the strongest bias towards CT codons, followed by *L. striatellus* and then Maize. Serine (Ser): All three hosts show a preference for the TCT codon over other Serine codons (TCC, TCA, TCG). Rice has the strongest bias towards TCT, followed by insect vectors and Maize. Arginine (Arg): All three hosts show a preference for the AGA codon over other Arginine codons (CGT, CGC, CGA, CGG*). L. striatellus* exhibits the strongest bias towards AGA, followed by Rice and Maize. Overall, the RSCU analysis reveals differences in codon usage preferences between the virus in rice, maize, and insect vectors. This suggests that the virus might have adapted its codon usage to the specific tRNA pool of each host for efficient translation. Furthermore, RBSDV is under negative or purifying selection, meaning mutations that disrupt essential functions are less likely to persist [19, 49]. ENC-plot and neutrality-plot analyses on two proteins P8 and P10 indicated that natural selection plays a major role in shaping the codon usage patterns of RBSDV and CAI analyses had a strong correlation between RBSDV and rice rather than other hosts (maize, wheat, or *Laodelphax striatellus*) [90]. While negative selection likely acts on most RBSDV fragments, the presence of numerous co-mutated hotspots across diverse populations suggests these mutations might confer an advantage to the virus. This advantage could explain the high frequency of these mutations in fragments under negative selection, allowing the virus to effectively spread across different host populations. Negative selection acts to eliminate deleterious mutations in viral proteins. These mutations disrupt essential functions and hinder the virus’s ability to replicate and spread. High-frequency mutations in a protein can seem contradictory to negative selection [91]. However, it is important to understand the nature of these mutations. In some cases, high-frequency mutations might represent escape mutations that allow the virus to evade the immune system, or to resist antiviral drugs or pesticides. These mutations can be beneficial in specific environments and would be under positive selection [92–95]. Therefore, the evolutionary pressure exerted by high-frequency mutations depends on the specific type of mutation.

This study aimed to identify the overall pool of mutations present in the transcriptomes of RBSDV populations from diverse hosts, including rice, maize, and insect vectors. Due to the variation in dataset collection, including different years, hosts, and regions, there is a possibility that some mutations (especially co-mutations) have become established within these specific viral populations. However, low-frequency mutations also deserve consideration. These mutations, though currently rare, could become more abundant and even dominant under certain environmental pressures. Overall, the presence of co-mutations within 3 years (2017–2020) suggests the RBSDV population is evolving. The specific implications depend on the type of mutation, selection pressures, and the virus itself.

## Conclusion

The RBSDV is a significant threat to the main food sources such as rice, maize, and other grain crops worldwide, leading to substantial economic losses. Originating in East Asian countries like China, the disease causes intermittent epidemics. In this study, we investigated the RBSDV transcriptomic populations through native *L. striatellus* and some plant hosts (RBSDV-susceptible/or normal) in China datasets, focusing on specific genome fragments and encoded proteins (P5-1, P5-2, P6, P7-1, P7-2, P9-1, P10) associated with pathogenicity and hosting. By analyzing viral proteins involved in transmission, formation of viroplasm, replication, assembly, and interaction with viral and plant factors, we identified forty-seven co-mutated hotspots with highly variable frequencies (HVF) in crucial regions. Among the RBSDV-infected populations, the RBSDV-susceptible rice cultivar KTWYJ3 and indigenous *L. striatellus* displayed the highest number of hotspots, with seven unique to *L. striatellus*. These findings suggest the insect vector’s high sensitivity and genetic diversity. Through a comprehensive survey, we discovered 2,646 single nucleotide polymorphisms (SNPs) and codon changes in the RBSDV whole transcriptome, highlighting numerous mutated hotspots in key proteins. Identical hotspots with high frequencies were prevalent in several RBSDV-infected host populations, indicating the rapid spread of co-mutated hotspots and the formation of dominant populations. Gene expression analysis revealed distinct patterns between plant hosts and the insect vector, suggesting correlations between specific genomic fragments and RBSDV actions in *L. striatellus*. Despite many unclear functions and interactions for RBSDV-encoded proteins, we propose that P5-2, P7-2, and P9-2 play vital roles in virus/planthopper interactions. Additionally, the mentioned genomic fragments in the planthopper showed higher specificity in hotspot mutations, potentially indicating increased mutational pressure in their crucial domains. Although some hotspots were identified in the most likely critical regions of the P7-1 genomic fragment, in future studies further examination with advanced tools is recommended. Moreover, the influence of host factors in the process of RBSDV evolution with a deeper examination in future studies seems necessary. Overall, our study unveils the extensive genetic diversity in RBSDV populations, which could lead to changes in the plant host and insect vector types, potentially expanding the host range and virulence evolution of RBSDV.

**Table 3:** Single-nucleotide polymorphisms (SNPs) among replicative populations of RBSDV in different plant and insect hosts

SNPs	Datasets	Hosts	Position	PE^∗^	VF (%)		Codon / Amino acid Change
R146.7HP5	D2-1, D2-2, RB MG1, RB MG3, RBSDV1, RBSDV2 and RBSDV3	Rice Planthopper	Overlapping P5-1 and P5-2 2817nt	SPE	100, 100, 100, 99.3, 99.3		CGU → CAU
M40.7TP5	D2-2, RB MG1, RB MG2, RBSDV1, RBSDV2 and RBSDV3	Rice Planthopper	Overlapping P5-1 and P5-2 2499nt	SPE	100, 100, 100, 97.5		AUG → ACG
P9.7LP5	D2-2, RBSDV1, RBSDV2 and RBSDV3	Rice Planthopper	Overlapping P5-1 and P5-2 2406nt	SPE	100, 8.8		CCA → CTA
S115.7NP5	D2-2, RB MG1, RB MG2, RB MG3, RBSDV1, RBSDV2 and RBSDV3	Rice Planthopper	Overlapping P5-1 and P5-2 2724nt	SPE	99 ,99.4, 99.4, 100, 93.2		AGC → AAC
P228.7LP5	RB MG1, RB MG2, RB MG3	Planthopper	P5-2 and 3′UTR 3063nt	SPE	100, 100, 100		CCU → CTU
A212.3TP5	RB MG1, RB MG2, RB MG3, RBSDV1, RBSDV2 and RBSDV3	Rice Planthopper	P5-2 and 3′UTR 3014nt	SPE	64.8, 73.8, 77.4, 2.7		GCU → ACU
T18.7IP5	RB MG1, RB MG2	Planthopper	Overlapping P5-1 and P5-2 2433nt	SPE	43.3 ,56.3		ACU → ATU
I651.3VP6	b73 t1, b73 t2^1^, D2-1, D2-2, RB MG1, RB MG2	Rice Maize Planthopper	P6, between amino acids 365 to 659	SPE	100, 23.8, 71.4, 100, 100, 27.7, 46.1		AUU → GUG AUU → GUG^1^
Y581.7CP6	b73 t1, D2-2, RB MG1, RB MG2, RB MG3	Rice Maize Planthopper	P6, between amino acids 365 to 659	SPE	100, 100, 41.3, 42.6, 43.8		UAC → UGC
V572.3IP6	b73 t1, RB MG3	Maize Planthopper	P6, between amino acids 365 to 659	SPE	100, 30.9		GUU → AUU
A545.3TP6	b73 t1, b73 t2, RB MG1, RB MG2	Maize Planthopper	P6, between amino acids 365 to 659	SPE	100, 19.7, 30.4, 37		GCU → ACC
N492.7TP6	b73 t1, RB MG1, RB MG2, RB MG3	Maize Planthopper	P6, between amino acids 365 to 659	SPE	100, 22.4, 37.2, 28.3		AAU → ACU
T583.3AP6	b73 t2, D2-2, RB MG1, RB MG2	Rice Maize Planthopper	P6, between amino acids 365 to 659	SPE	100, 100, 100, 100		ACA → GCA
N516.3DP6	b73 t2, D2-2, RB MG1, RB MG2, RBSDV1, RBSDV2 and RBSDV3	Rice Maize Planthopper	P6, between amino acids 365 to 659	SPE	92, 100, 26.7, 33.8, 78.6		AAC → GAC
Y429.3PP6	b73 t2, D2-2, RB MG1, RB MG2	Rice Maize Planthopper	P6, between amino acids 365 to 659 and 404 to 439	SPE	78.6, 99.4, 37.1, 37.3		UAU → CCU
F518.3LP6	b73 t2, D2-2, RB MG1, RB MG2	Rice Maize Planthopper	P6, between amino acids 365 to 659	SPE	77.3, 86.3, 30.8, 26.9		UUU → CUU
S509.3IP6	b73 t2, D2-1, D2-2, RB MG1, RB MG2, RB MG3	Rice Maize Planthopper	P6, between amino acids 365 to 659	SPE	75, 100, 100, 32.4, 40.4, 64.4		UCU → ATU
S473.3AP6	b73 t2, D2-2, RB MG1, RB MG2, RBSDV1, RBSDV2 and RBSDV3	Rice Maize Planthopper	P6, between amino acids 365 to 659	SPE	69.6, 100, 100, 27.5, 31.9, 83.2		UCU -> GCU
N510.3DP6	D1, RB MG1, RB MG2	Rice Planthopper	P6, between amino acids 365 to 659	SPE	100, 10.8, 7.9		AAU -> GAU
G587.7DP6	D2-2, RB MG1, RB MG2	Rice Planthopper	P6, between amino acids 365 to 659	SPE	100, 58.7, 64.5		GGU -> GAC GGU -> GAU^3^
Q519.7LP6	D2-2, RB MG1, RB MG2	Rice Planthopper	P6, between amino acids 365 to 659	SPE	99.1, 30.8, 28.6		CAG -> CTG
S458.7NP6	D2-2, RB MG1, RB MG2	Rice Planthopper	P6, between amino acids 365 to 659	SPE	98.8, 39.4, 35.2		AGU -> AAU
N656.7TP6	RB MG1, RB MG2	Planthopper	P6, between amino acids 365 to 659	SPE	69.2, 50.8		AAU -> ACU
Y429.7SP6	RB MG1, RB MG2, RB MG3	Planthopper	P6, between amino acids 365 to 659 and 404 to 439	SPE	62.9, 62.7, 77.1		UAU -> UCU
A655.7VP6	RB MG1, RB MG2, RB MG3, RBSDV1, RBSDV2 and RBSDV3	Rice Planthopper	P6, between amino acids 365 to 659	SPE	31.3, 48.9, 24.6, 78.6		GCU -> GTU
R294.3GP7	b73 t1	Maize	P7-1, TM2 region, between amino acids 286 to 303	SPE	66.70		AGA -> GGA
F108LP7	b73 t1	Maize	P7-1, TM1 region, between amino acids 108 to 126	SPE	50.00		UUU -> UUG
K302.7RP7	D2-1, MG 1	Rice, Planthopper	P7-1, TM2 region, between amino acids 286–303	SPE	37.50, 4.3		AAA -> AGA
I301.7TP7	D2-1	Rice	P7-1, TM2 region, between amino acids 286 to 303	SPE	37.50		AUU -> ACU
L300.3MP7	D2-1	Rice	P7-1, TM2 region, between amino acids 286 to 303	SPE	37.50		UUG -> AUG
I298.7NP7	D2-1	Rice	P7-1, TM2 region, between amino acids 286 to 303	SPE	33.30		AUC -> AAC
V297.3IP7	D2-1	Rice	P7-1, TM2 region, between amino acids 286 to 303	SPE	33.30		GUC -> AUC
Q112.7LP7	MG 3	Planthopper	P7-1, TM1 region, between amino acids 108 to 126	SPE	6.60		CAA -> CTA
N123.3DP7	RBSDV1 RBSDV2 RBSDV3	Rice	P7-1, TM1 region, between amino acids 108 to 126	SPE	74.2		AAU -> GAC
T294.3AP7	RBSDV1 RBSDV2 RBSDV3	Rice	P7-1, TM2 region, between amino acids 286 to 303	SPE	57.10		ACC -> GCC
E293.3KP7	b73 t2, D2-2, RB MG1, RB MG2	Rice Maize Planthopper	P7-2, between amino acids 1 to 295	SPE	100, 100, 100, 100		GAG -> AAG
I166.3LP7	b73 t2, D1, D2-2, RB MG1, RB MG2, RBSDV1, RBSDV2 and RBSDV3	Rice Maize Planthopper	P7-2, between amino acids 1 to 295	SPE	100, 100, 99.3, 99.3, 99.8, 99.2		AUA -> TUA
R52.3CP7	b73 t2, D1, D2-2- RB MG1, RB MG2	Rice Maize Planthopper	P7-2, between amino acids 1 to 78 and 1 to 295	SPE	100, 100, 100, 100, 100		CGU -> TGU
G5.3SP7	b73 t2, D2-2- RB MG1, RB MG2	Rice Maize Planthopper	P7-2, between amino acids 1 to 78 and 1 to 295	SPE	100, 100, 99.2, 98.9		GGU -> AGU
Q288.7RP7	D2-2, RB MG1, RB MG2	Rice Planthopper	P7-2, between amino acids 1 to 295	SPE	100, 100, 98.6		CAA -> CGA
N149.7SP7	RB MG1, RB MG2, RB MG3	Planthopper	P7-2, between amino acids 1 to 295	SPE	90.6, 94, 96.7		AAU -> AGU
S195.3RP7	RB MG1, RB MG2	Planthopper	P7-2, between amino acids 1 to 295	SPE	84.3, 87.6		AGU -> CGU
P168SP9	RB MG1, RB MG2, RB MG3, D2-2, RBSDV1, RBSDV2, RBSDV3, b73 t2	Rice Maize Planthopper	P9-1, between amino acids 1 to 347	SPE	100, 99.6, 100, 99.7, 99.4, 99.3, 100		Pro -> Ser
G84AP9	RB MG1, RB MG2, RB MG3, D2-2, RBSDV1, RBSDV2, RBSDV3, b73 t2	Rice Maize Planthopper	P9-1, between amino acids 1 to 347	SPE	69.1, 59.4, 64.6, 100, 55.5, 70.5, 74.4, 100		Glu -> Asp
L20AP9	RB MG1, RB MG2, RB MG3, D2-2, RBSDV1, RBSDV2, RBSDV3, b73 t2	Rice Maize Planthopper	P9-1, between amino acids 1 to 347	SPE	59.1, 56.4, 69, 99.35, 82.1, 83.4, 64.4, 18.18		Lys -> Arg
T32AP9	RB MG1, RB MG2, RB MG3, D2-2, RBSDV1, RBSDV2, RBSDV3, b73 t2	Rice Maize Planthopper	P9-1, between amino acids 1 to 347	SPE	60, 57.8, 66.4, 100, 82, 84.6, 71.7, 15		Thr -> Asn
V151AP9	RB MG1, RB MG2, RB MG3, D2-2, RBSDV1, RBSDV2, RBSDV3, b73 t2	Rice Maize Planthopper	P9-1, between amino acids 1 to 347	SPE	66.7, 71.13, 75, 100, 73.2, 70, 66.3		Val -> Ala
T295AP9	RB MG1, RB MG2, RB MG3, D2-2, RBSDV1, RBSDV2, RBSDV3, b73 t2	Rice Maize Planthopper	P9-1, between amino acids 1 to 347	SPE	73, 69.5, 80.3, 99.5, 83.29, 84.5, 9.13, 17		Thr -> Ala
A104AP9	RB MG1, RB MG2, RB MG3, D2-2, RBSDV1, RBSDV2, RBSDV3, b73 t2	Rice Maize Planthopper	P9-1, between amino acids 1 to 347	SPE	57.3, 47.2, 51.5, 100, 93.5, 87, 82.3, 16		Asp -> Asn
P137SP9	RB MG1, RB MG2, RB MG3, D2-2, RBSDV1, RBSDV2, RBSDV3, b73 t2	Rice Maize Planthopper	P9-1, between amino acids 1 to 347	SPE	60.4, 61, 66, 99.6, 75, 80.6, 68, 11.5		Pro -> Ser
T138PP9	RB MG1, RB MG2, RB MG3, D2-2, RBSDV1, RBSDV2, RBSDV3	Rice Maize Planthopper	P9-1, between amino acids 1 to 347	SPE	65, 65.6, 69.5, 99.8, 75, 71, 68.1		Thr -> Pro
C256.3SP10	b73 t2, D2-1, D2-2, RB MG1, RB MG2	Rice Maize Planthopper	P10, TM2, between amino acids 250 to 270	SPE	100, 100, 100, 96, 98.3		UGC -> AGC
L124.3FP10	b73 t2, D1, D2-1, D2-2, RB MG1, RB MG2	Rice Maize Planthopper	P10, TM1, between amino acids 119 to 137	SPE	94.4, 100, 100, 100, 100, 99.6		CUU -> TUU
S68.7LP10	D2-1, D2-2, RB MG1, RB MG2,	Rice Planthopper	P10, between amino acids 1 to 270	SPE	100, 99.3, 100, 99.7		UCG -> UTG
F108.0LP10	RB MG3, RBSDV1, RBSDV2 and RBSDV3	Rice Planthopper	P10, between amino acids 1 to 270	SPE	100, 99.6		UUU -> UUG
Y88.7CP10	RB MG3, RBSDV1, RBSDV2 and RBSDV3	Rice Planthopper	P10, between amino acids 1 to 270	SPE	58.9, 58.1		TAC -> TGC

∗ The protein effects (PE), The substitution protein effects (SPE), variant frequency (VF%), The amino acids: Proline (Pro), Serine (Ser), Glutamic acid (Glu), Aspartic acid (Asp), Lysine (Lys), Arginine (Arg), Threonine (Thr), Asparagine (Asn), Valine (Val), Alanine (Ala)

### Electronic supplementary material

Below is the link to the electronic supplementary material.

Supplementary Material 1

Supplementary Material 2

Supplementary Material 3

Supplementary Material 4

## Data Availability

The raw RNA-seq data are available in the NCBI database with the Accession Numbers SRX2653517, SRX2730361-SRX2730362, SRX8967824-SRX8967826, SRX3785263-SRX3785264, and SRX8604131- SRX8604133. All data generated or analyzed during this study are included in this published article and supplementary information files 1, 2 and 3.
